# Medication overuse headache is associated with elevated lipopolysaccharide binding protein and pro-inflammatory molecules in the bloodstream

**DOI:** 10.1186/s10194-023-01672-4

**Published:** 2023-11-08

**Authors:** Hale Gök Dağıdır, Elif Topa, Doga Vuralli, Hayrunnisa Bolay

**Affiliations:** 1 Neuroscience and Neurotechnology Center of Excellence (NÖROM), Gazi University, Beşevler, Ankara, Türkiye; 2 Neuropsychiatry Center, Gazi University, Beşevler, Ankara, Türkiye; 3 Department of Neurology and Algology, Faculty of Medicine, Gazi University, Beşevler, Ankara, Türkiye

**Keywords:** MOH, Migraine, Leaky gut, LPS, LBP, HMGB1, Occludin, VE–cadherin, IL-17, Inflammation

## Abstract

**Objective:**

Medication overuse headache (MOH) is a secondary headache that accompanies chronic migraine. Nonsteroidal anti-inflammatory drugs (NSAIDs) are the most frequently used analgesics worldwide and they are known to induce leaky gut. In this study, we aimed to investigate whether NSAID induced MOH is associated with altered circulating lipopolysaccharide binding protein (LBP) levels and inflammatory molecules.

**Materials and methods:**

Piroxicam (10 mg/kg/day, po) for 5 weeks was used to induce MOH in female Sprague Dawley rats. Pain behavior was evaluated by periorbital withdrawal thresholds, head-face grooming, freezing, and head shake behavior. Serum samples and brain tissues were collected to measure circulating LBP, tight junction protein occludin, adherens junction protein vascular endothelial (VE)-cadherin, calcitonin gene-related peptide (CGRP), IL-6 levels and brain high mobility group box-1 (HMGB1) and IL-17 levels.

**Results:**

Chronic piroxicam exposure resulted in decreased periorbital mechanical withdrawal thresholds, increased head-face grooming, freezing, and head shake behavior compared to vehicle administration. Serum LBP, CGRP, IL-6, IL-17, occludin, VE-cadherin levels and brain IL-17 and HMGB1 levels were significantly higher in piroxicam group compared to controls. Serum LBP was positively correlated with occludin (r = 0.611), VE-cadherin (r = 0.588), CGRP (r = 0.706), HMGB1 (r = 0.618) and head shakes (r = 0.921), and negatively correlated with periorbital mechanical withdrawal thresholds (r = -0.740).

**Conclusion:**

Elevated serum LBP, VE-cadherin and occludin levels indicating disrupted intestinal barrier function and leakage of LPS into the systemic circulation were shown in female rats with MOH. LPS induced low-grade inflammation and elevated nociceptive and/or pro-inflammatory molecules such as HMGB1, IL-6, IL-17 and CGRP may play a role in the development and maintenance of MOH. Interference with leaky gut and pro-inflammatory nociceptive molecules could also be a target for sustained management of MOH.

## Introduction

Medication overuse plays an important role in the transformation of episodic headaches into chronic headache disorders, thus resulting in a vicious circle with more drug consumption and increased headache frequency with treatment-resistant chronic headache. In this case, the treatment of the headache attacks becomes a trigger for headaches and medication overuse headache (MOH) develops [1, 2].

MOH is a chronic headache disorder that is more prevalent in women (approximately 75% of the patients) [3] and occurs secondary to analgesic abuse, mostly non-steroidal anti-inflammatory drugs (NSAIDs). NSAIDs are commonly used to abort headache attacks. These drugs show their effects by inhibiting the activity of cyclooxygenase, which catalyzes the production of prostaglandins responsible for pain and inflammation [4]. However, NSAIDs have disadvantages as they are toxic to the gastric and intestinal epithelium and may disrupt barrier function in the gastrointestinal (GI) system [5]. Injury to the intestinal epithelial cells and/or disrupted gut vascular barrier integrity can alter intestinal permeability leading to leaky gut. Diclofenac, in a physiological dose, was shown to increase intestinal permeability by interfering with mitochondrial oxidative phosphorylation in human intestinal epithelial cells, releasing reactive oxygen species and disrupting tight junctions [5]. Thus, long-term use of pharmacologic agents such as NSAIDs can increase intestinal permeability and elicit inflammatory response [6, 7].

The intestinal barrier consists of epithelial cells connected with each other through desmosomes, adherens junctions, and tight junctions. When intestinal wall structures are disrupted, barrier functioning is impaired and a leakage of toxic substances and lipopolysaccharides (LPS) into the systemic circulation occurs [8]. Serum LPS level increases in leaky gut [8]. LPS, bacterial endotoxin, is a part of the outer membrane of Gram-negative bacteria that are primarily found in intestinal lumen. The presence of LPS in the systemic circulation triggers low grade inflammatory response in the host. LPS binding protein (LBP) is an acute phase protein secreted by liver in response to LPS leakage into the bloodstream. LBP binds to LPS exclusively and LBP concentration is positively correlated with LPS levels in the circulation [9, 10].

The aim of our study is to investigate whether leaky gut is associated with MOH. Even though the role of gut-brain axis is emphasized in migraine [11], there is no preclinical or clinical study proving the concept of leaky gut in headache disorders with objective serum parameters. There is only one pain study that has shown higher LBP levels associated with abnormal pain thresholds from intraepidermal electrical stimulation in the general Japanese population [12]. MOH models induced by NSAIDs in the literature are few [13, 14], and we used chronic piroxicam overuse to simulate MOH in rats. Considering the high prevalence of MOH and higher impact of NSAID induced disturbance in women, the model was designed in female Sprague Dawley rats [3, 15]. Similarly, there are previous MOH models studied in merely female rodents [16]. We decided to investigate the crucial components of intestinal barrier, tight junction, and adherens junction proteins, occludin and vascular endothelial cadherin (VE-cadherin) respectively.

Another aim was to study several critical pro-inflammatory and/or nociceptive molecules, since LPS leak into the systemic circulation elicits low grade inflammation. We also assessed serum calcitonin gene-related peptide (CGRP) level, a key molecule in migraine pathophysiology and trigeminal nociception. Interleukin-6 (IL-6) is both a pro-inflammatory and nociceptive molecule, that is involved in various headache models [17] and migraine patients [18]. Pro-inflammatory cytokine IL-17 is pivotal for immune regulation in the gut and was shown to be involved in the central nervous system in nitroglycerin (NTG) migraine model [19]. Therefore, we included IL-17 in our MOH study. High-mobility group box 1 (HMGB1) is a master regulator in innate immunity and implicated in preclinical headache models and secondary headaches [20–22]. HMGB1 and LPS act through the same receptors, toll like receptor-4 (TLR-4), and synergistically amplify inflammatory response.

## Materials and methods

### Animals and experimental groups

Female Sprague Dawley rats weighing 200-250gr were used in experiments. Rats were housed in a climate-controlled environment with alternating periods of twelve hours of dark and twelve hours of light. Rats were free to access water and food.

**Fig. 1:**
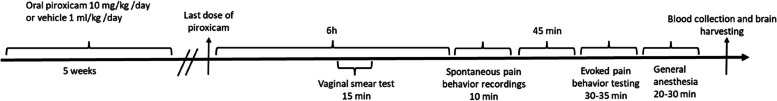
Experimental design of the study

In the study, 16 female Sprague Dawley rats (8 vehicle and 8 piroxicam) were used. Oral piroxicam (10 mg/kg/day) was given daily for 5 weeks by gavage to induce MOH. Control group rats were given the same amount of vehicle (water, 1 ml/kg/day) for the same period. Ethical approval was obtained from Gazi University Animal Studies Ethical Committee and the study was conducted according to the National guidelines for the care and use of laboratory animals. Behavioral testing was performed according to estrous cycle staging. The experimental design is given in Fig. 1.

### Behavioral studies

Estrous cycle staging was performed by vaginal cytology in female rats. The behavioral tests were not performed during the pro-estrous and estrous phases since pain thresholds are reduced in those estrous cycle stages [23]. Behavioral tests were performed at the same time frame of the day. Pain related spontaneous behavior such as head and face grooming, freezing [24], and head shakes were recorded for 10 min in a plexiglass cage with a video-camera system set-up. Mechanical withdrawal thresholds were evaluated with von Frey filaments using a set of calibrated von Frey filaments ranging from 0.008 g to 15 g (Aesthesio® Precision Tactile Sensory Evaluators) and “up-and-down” method was used starting from 2.0 g, applied to the mid-rostral portion of the eyes [25]. Averting response, head shakes or grooming were counted as a positive response. 50% withdrawal thresholds were calculated using the free online calculator at https://bioapps.shinyapps.io/von_frey_app/.

### ELISA

Following the last behavioral testing, rats were anesthetized by a mixture of intraperitoneal (ip) ketamine (Ketalar, Pfizer, 60 mg/kg) and xylazine (RompunTM, Bayer, 8 mg/kg), intracardiac blood was taken, and cortical brain tissues were harvested. Serum was obtained by centrifugation (20 min 1000*g at 2–8 °C) in accordance with the ELISA (Enzyme-Linked Immuno Sorbent Assay) kit protocol. Serum and brain cortex samples were stored at − 80 °C until analyzed. Serum LBP, CGRP, IL-17, IL-6, occludin, VE-cadherin levels and brain HMGB1 and IL-17 levels were measured by high-sensitivity ELISA Rat Kits. The commercial kits were used according to their instructions. Sandwich-type Rat ELISA kits were obtained from Elabscience Biotechnology Inc., Houston, TX, USA [Serum LBP (E-EL-R0589), CGRP1 (E-EL-R0135), IL-17 (E-EL-R0566), IL-6 (E-EL-R0015), Occludin (E-EL-R2503), VE-cadherin (E-EL-R0130) and brain tissue HMGB1 (E-EL-R0505)] and MyBioSource Inc., USA [brain tissue IL-17 (MBS2022678)]. The coefficients of variation for the repeatability were < 10%.

Before analysis, cortical brain tissues were homogenized in T-PER TM Tissue Protein Extraction Reagent (w:v = 1:20 in 1 ml buffer for 20 mg tissue sample) on ice (pH = 7.6), Thermo Scientific Halt TM Protease Inhibitor Cocktail EDTA Free 78,437 was added (10 μL concentrated cocktail per 1 mL lysis buffer) to protect cellular proteins from degradation by endogenous proteases and then centrifuged at 10,000*g for 5 min at 2–8 °C. Standards and samples were carefully studied in duplicate. The solutions used in the kits were prepared as specified in the ELISA kit procedure and analytical grade deionized water was used to prepare the solutions. All reagents were brought to room temperature before use as specified in the analysis procedure. Standards and samples were added to the wells on the plate in the desired microliters in each kit. The addition order of the solutions, the number of washes and the incubation times were progressed in accordance with the procedure of each kit. In the incubations, the plates were covered with a sealing film. After the last wash tetramethylbenzidine was added to each well to achieve a blue color reaction. The optical density of each well was measured using a microplate reader set to 450 nm, with a rapid change of color from blue to yellow after adding an acidic stop solution to each well.

Serum and tissue homogenate concentrations studied by ELISA method were calculated according to the standard graph. During the analysis, Combiwash Human ELISA plate washer and Chromate reader were used.

### Statistics

Experiments were performed and results were analyzed in a blinded fashion. Statistical analysis of the data was performed by the SSPS Statistics software, version 25.0. Sample size for each group was calculated using G*power [26]. From preliminary experiments, we have determined an effect size of 1.4. Type I error (α error) probability and type II error (β error) were set at 0.05 and 0.8 respectively. These values provided a total sample size of 16, with 8 animals in each experimental group. A total of 16 animals was used during this study. Normal distribution of data was evaluated with the Kolmogorov–Smirnov test. The analysis of continuous variables was performed using student t-tests (parametric), Mann–Whitney’s U test (non-parametric) or 2-way analysis of variance for repeated measures when there were repeated measures. Post-hoc multiple comparisons were performed using Šidák’s multiple comparisons test. Statistical significance level was accepted as *p* < 0.05. All statistical tests were two-sided. Continuous variables were given as mean ± standard deviation when Student’s t test was used and as median (Q1-Q3) and confidence interval when Mann–Whitney’s U test was used. Spearman correlation method was used and a p value less than 0.05 was considered statistically significant. The direction and magnitude of the relationship between the independent variables were indicated by the correlation coefficient (r) (a value between (-1) and (+ 1)).

## Results

**Fig. 2:**
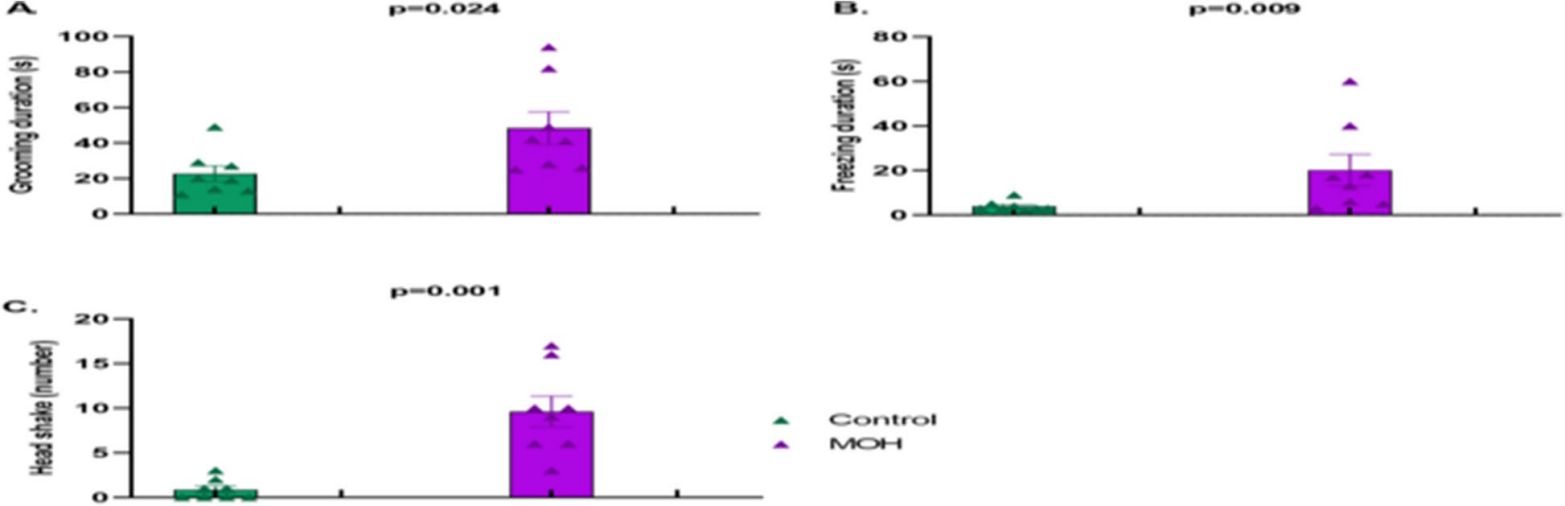
Behavioral results of female Sprague Dawley rats exposed to chronic piroxicam (MOH) or vehicle (Control). Compared to the control group, **A** the total duration of grooming, **B** total duration of freezing and **C** the number of head shakes were significantly higher in MOH group. Data were shown as mean ± SEM. Normality of data was evaluated with the Kolmogorov–Smirnov test. Statistical analysis was performed with Mann–Whitney’s U test

**Fig. 3:**
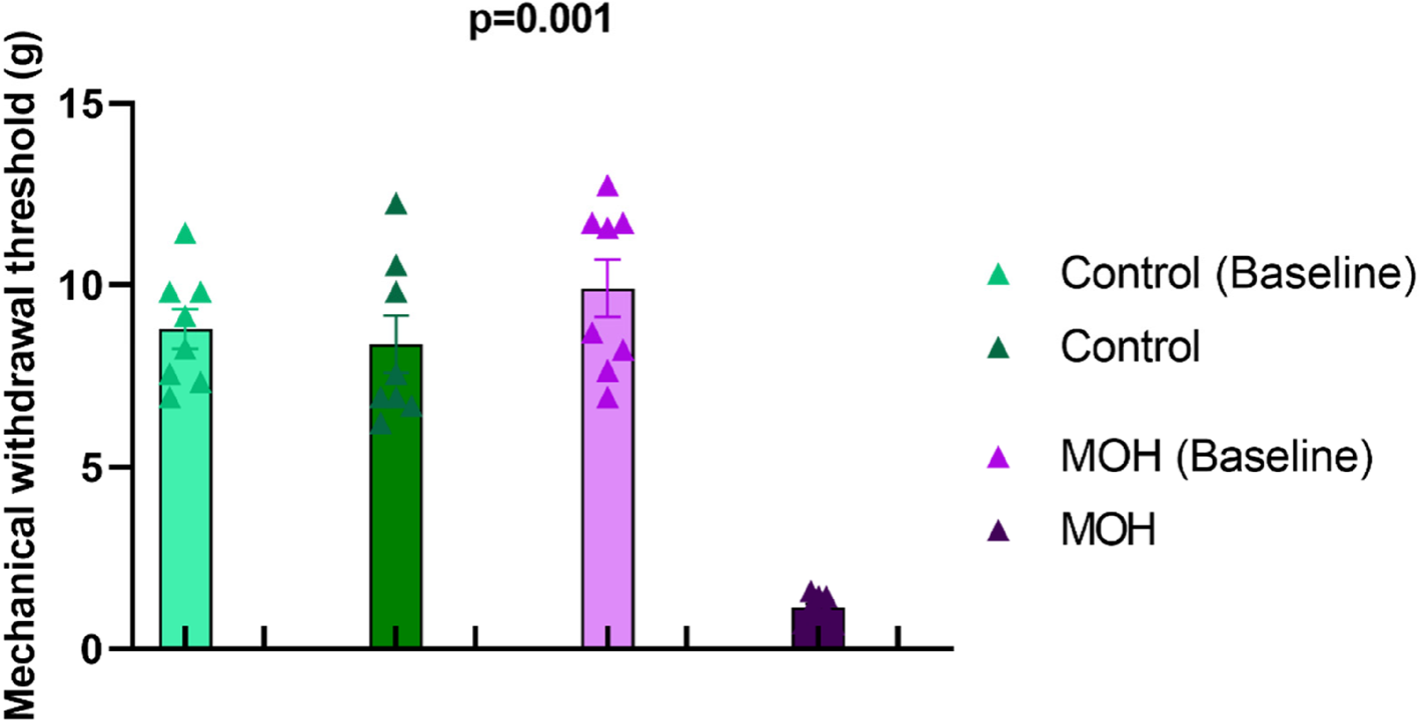
Mechanical withdrawal thresholds of female Sprague Dawley rats exposed to chronic piroxicam (MOH) or vehicle (Control). The mechanical withdrawal thresholds were significantly lower in MOH group. Data were shown as mean ± SEM. Statistical analysis was performed with 2-way analysis of variance for repeated measures. Post-hoc multiple comparisons were performed using Šidák’s multiple comparisons test

Animal well-being was maintained during a 5-week period of drug or vehicle administration without any complication or mortality. After 5 weeks of exposure to piroxicam or vehicle, the total duration of head-face grooming behavior (*p* = 0.024), the total duration of freezing (*p* = 0.009), and the number of head shakes (*p* = 0.001) were significantly higher in the chronic piroxicam group compared to its vehicle (Fig. 2). Mechanical withdrawal thresholds in the periorbital area were also significantly lower in chronic oral piroxicam group compared to vehicle group (*p* = 0.001) (Fig. 3).

**Fig. 4:**
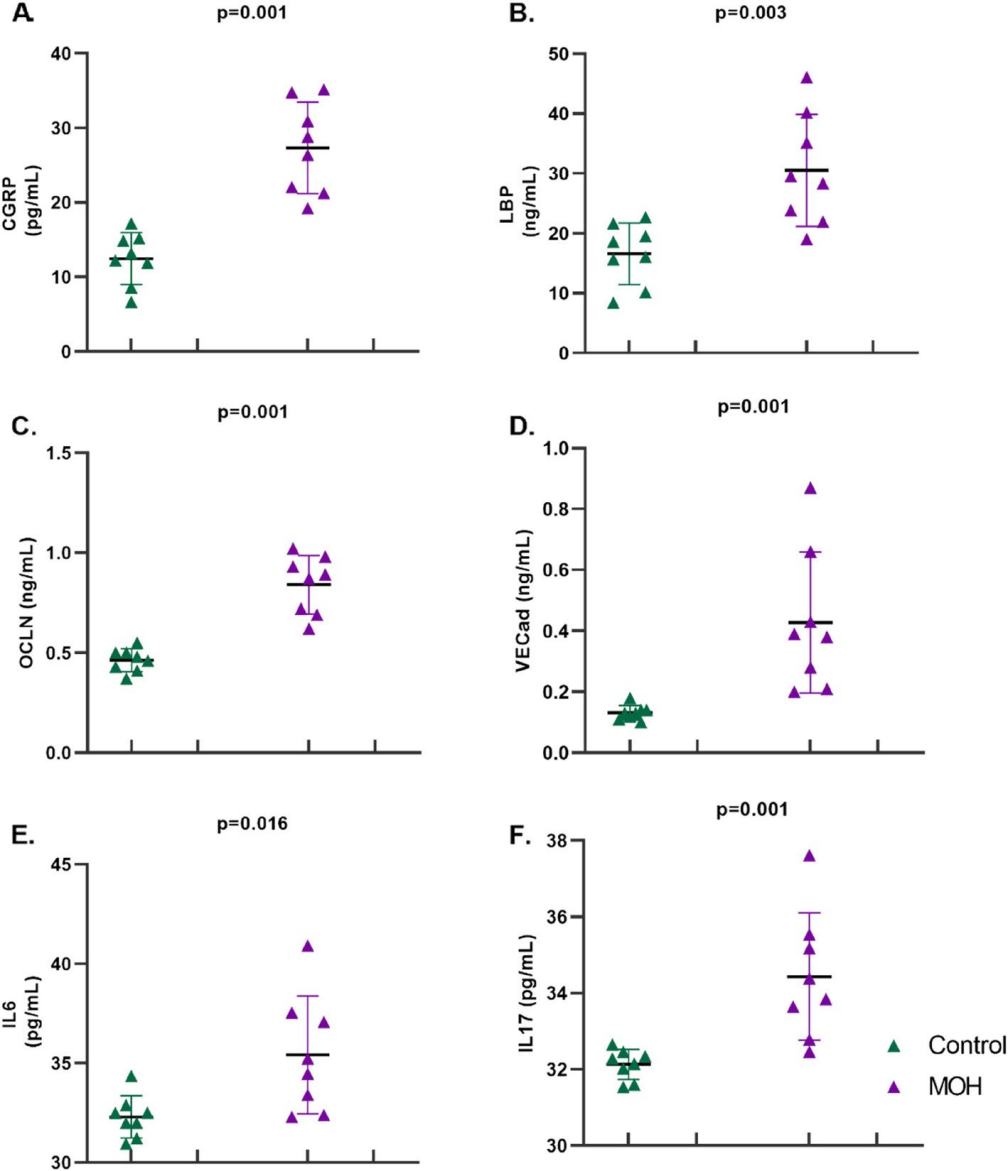
Serum levels of circulating biomarkers of leaky gut, inflammation and pain in female Sprague Dawley rats exposed to chronic piroxicam (MOH) or vehicle (Control). Compared to the control group, serum levels of **A** CGRP, **B** LBP, **C** occludin, **D** VE-cadherin, **E** IL-6 and **F** IL-17 levels were significantly higher in MOH group compared to the control group. Data were shown as mean ± SD. Statistical analysis was performed with student t-test for CGRP, LBP and occludin and Mann–Whitney’s U test for VE-cadherin, IL-6 and IL-17

Serum samples were measured with the ELISA method and compared within groups. Serum CGRP (27.31 ± 6.13 vs 12.46 ± 3.49 *p* = 0.001), LBP (30.51 ± 9.34 vs 16.58 ± 5.13 *p* = 0.003), occludin (0.84 ± 0.15 vs 0.46 ± 0.06 *p* = 0.001), VE-cadherin (median 0.38 CI (0.23–0.60) vs 0.128 (0.11–0.14), *p* = 0.001), IL-6 (median 34.85 CI (32.64–37.42) vs 32.24 (31.41–32.79), *p* = 0.016), IL-17 (median 34.11 CI (32.99–35.45) vs 32.21 (31.70–32.43), *p* = 0.001) levels were significantly higher in piroxicam received rats (MOH) compared to controls (Fig. 4).

CGRP, LBP, occludin, VE-cadherin, IL-6, and IL-17 levels were significantly higher in MOH group compared to the control group.

**Fig. 5:**
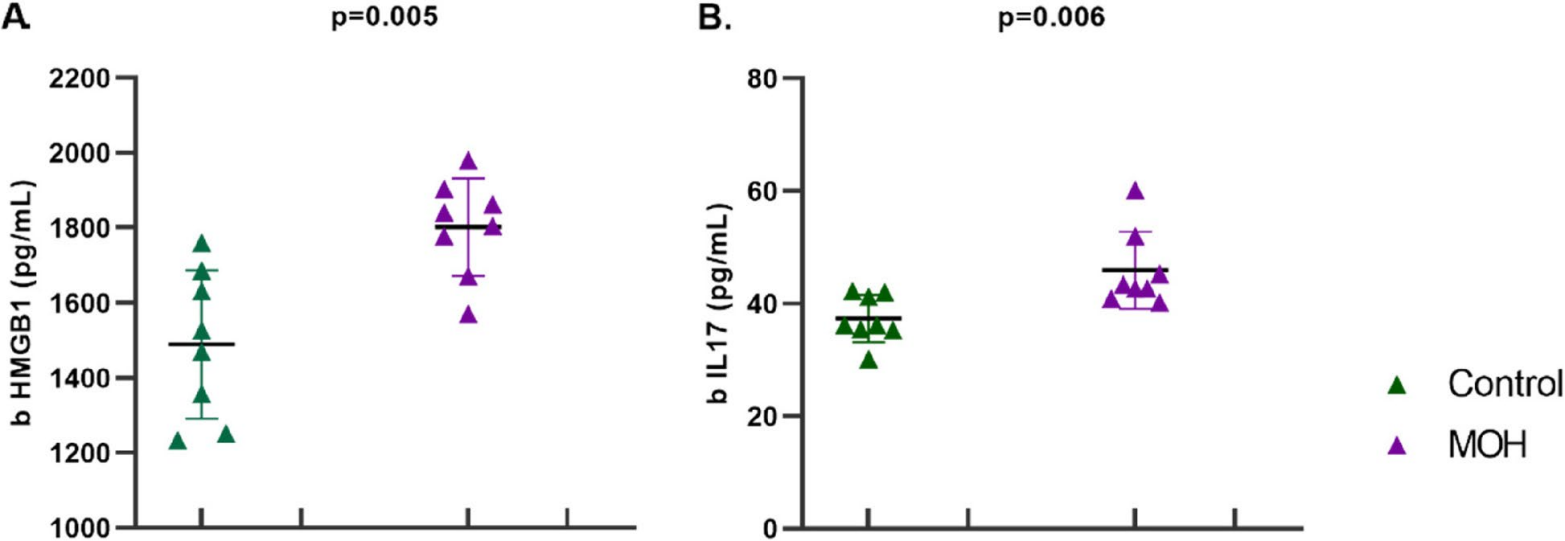
Cerebral cortical levels of inflammatory molecules of female Sprague Dawley rats exposed to chronic piroxicam (MOH) or vehicle (Control). Compared to the control group, cortical **A** HMGB1 and **B** IL-17 levels were significantly higher in MOH group. Data were shown as mean ± SD. Statistical analysis was performed with student t-test

Five weeks of chronic piroxicam administration increased inflammatory molecules in cerebral cortical brain tissue in female Sprague Dawley rats. Cortical IL-17 (45.90 ± 6.81 vs 37.35 ± 4.18 *p* = 0.006) and HMGB1 (1801.12 ± 129.95 vs 1489.33 ± 197.35 *p* = 0.005) levels were significantly higher in MOH group compared to the controls. The results are shown in Fig. 5.

Serum LBP level was positively correlated with serum occludin (r = 0.611), CGRP (r = 0.706), VE-cadherin (r = 0.588) and brain HMGB1 (r = 0.618) levels Table 1.

**Table 1:**
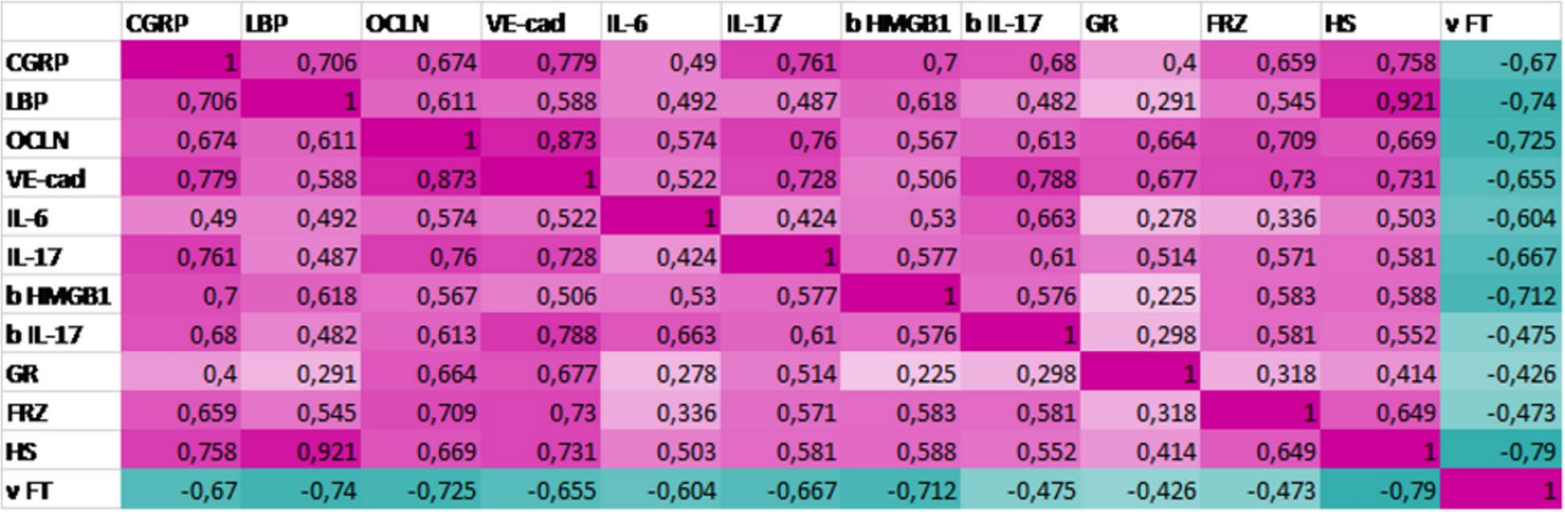
Correlation matrix shows the interrelationship between biomarkers [inflammatory parameters, CGRP, LBP, occludin (OCLN), VE-cadherin (VE-cad)] and pain behavior [total head-face grooming-GR, freezing-FRZ, head shakes-HS and mechanical withdrawal thresholds to von Frey filaments-vFT)

## Discussion

The present study provided evidence for the link between MOH and leaky gut by demonstrating an elevated occludin and VE-cadherin levels in the systemic circulation, increased LBP levels indicating LPS leak into the bloodstream and a significant inflammatory response in piroxicam induced MOH model. Five-week exposure to oral piroxicam elicited pain related behavior consistent with MOH. Reduced periorbital mechanical withdrawal thresholds, increased head and face grooming, freezing, head shake behavior and increased serum CGRP levels were compatible with trigeminal pain in the presented MOH model in female rats.

Similar to our study, spontaneous and evoked pain behavior have been shown in MOH animal models. Four-week mefenamic acid exposure has been shown to result in decreased periorbital mechanical withdrawal thresholds, elevated number of head shakes, freezing and grooming in rats [13]. Eleven day sumatriptan injection triggered mechanical allodynia in both periorbital regions and paws in mice [27]. Repeated sumatriptan injection induced activation of microglial cells and increased purinergic receptor P2X7 (P2X7R) expression and activation of NOD-, LRR- and pyrin domain-containing protein 3 (NLRP3) inflammasome in trigeminal nucleus caudalis [27] and inhibition of P2X7R and NLRP3 inflammasome attenuated sumatriptan induced mechanical allodynia [27]. Six doses of lasmitidan resulted in bright light stress or nitric oxide induced cutaneous allodynia in the periorbital and hindpaw regions [28].

Elevation of LBP levels which is a reliable biomarker of increased LPS in the bloodstream along with tight junction protein occludin and adherens junction component VE-cadherin suggests a disruption in the intestinal integrity and permeability change leading to leaky gut. The increased levels of junctional proteins can be detected in vascular permeability changes associated with BBB disruption and parenchymal damage [29, 30]. VE-cadherin also serves as a biomarker for gut-vascular barrier integrity, that limits passage of intestinal toxins into bloodstream [31]. In our study, LBP, occludin and VE-cadherin levels were positively correlated with each other. Also, serum VE-cadherin and occludin levels showed strong correlations with inflammatory cytokines, serum CGRP levels and pain behaviors. These findings indicated that trigeminal nociceptive responses and headache related behavior were correlated with intestinal barrier disruption and inflammatory responses.

The crucial role of gut-brain axis in neurological disorders including migraine headache has been acknowledged. There is a bidirectional relationship between gut permeability and inflammation [11]. Increased gut permeability stimulates inflammatory response in the host by leakage of LPS into the bloodstream. The induction of inflammatory cascade and release of pro-inflammatory cytokines upon LPS exposure further increases gut permeability [11]. Several pro-inflammatory cytokines such as IL-6 and IL-1β are also nociceptive in the trigeminovascular system and take a part in the headache development [32]. Disrupted intestinal barrier and associated inflammation has not been studied in MOH. However, in a translational NTG migraine model, antibiotic administration induced prolonged pain was blocked by tumor necrosis factor alpha (TNF-α) targeted interventions [33]. Increased NTG provoked pain in germ free mice suggested a role of microbiome in headache disorders [33].

Gut barrier integrity has gained importance in diverse systemic and neurological diseases [25, 26, 31]. Diagnostic tests to measure intestinal mucosal injury are challenging for the patients, therefore, potential blood biomarkers are used for indirect assessment of intestinal barrier function [34]. Bacterial endotoxins must be strictly kept in intestinal lumen, and the detection of LPS in the bloodstream indicates intestinal hyperpermeability and leaky gut. The latter is supported by the detection of elevated serum levels of tight/adherens junction proteins that are components of the intestinal barrier. In line with this notion, increased blood level of LPS was detected in patients with irritable bowel syndrome (IBS) [32].

LPS in the systemic circulation is recognized and bound by a soluble acute phase protein called LBP [35, 36]. A significant increase in serum LBP levels in MOH group was consistent with the presence of LPS in the blood circulation. LBP has been shown to be a reliable biomarker for the presence of LPS in the circulation and the assessment of intestinal permeability with its longer half-life and low intraindividual variability, independent of age, sex, and body-mass index [34, 37].

There is also a sexual dimorphism in intestinal permeability and women are more vulnerable to perturbations in intestinal permeability by NSAIDs [15]. The disturbance in intestinal permeability usually recovers 4–6 weeks after discontinuation of NSAID [15]. The latter may be one of the mechanisms underlying the clinical observation of headache improvement starting 1–2 months after the cessation of the analgesics in MOH patients [1, 2].

Intercellular tight junctions and adherens junctions are important structures for building the epithelial barrier [38, 39] and leaky gut syndrome occurs with loosening of these structures in the intestinal wall [39, 40]. Occludin is a key tight junction protein and serum occludin level can be a surrogate biomarker for intestinal permeability and gut disorders as well as BBB disruption in neurological disorders [29, 30]. VE-cadherin, an adherens junction molecule connecting cells is involved in the regulation of paracellular permeability [41]. The breakdown of endothelial adherens junction proteins is associated with disruption of vascular integrity in various organs [42]. Serum VE-cadherin was shown to be significantly elevated in severe sepsis [43]. Thereby we have proof that not only LPS is present in the blood circulation but also both tight junction and adherens junction structures are disrupted and occludin and VE-cadherin levels are increased in the blood circulation in MOH. All these findings indicate the presence of leaky gut in our MOH model.

LPS when present in the circulation in low dose, called metabolic endotoxemia is associated with metabolic disorders such as diabetes mellitus and obesity [44]. LPS elicits inflammatory response by activating TLR-4 receptors. Interestingly the same receptor can also be activated by HMGB1 [20]. Both LPS and HMGB1 are critical actors in innate immune response [45]. Upon cell activation or cell damage, HMGB1 translocates from the nucleus to the cytoplasm and is released into extracellular space. HMGB1 is a potent pro-inflammatory molecule and induces nociception through its TLR4 or RAGE receptors in the trigeminal neurons [46]. LPS and HMGB1 have a mutual interaction that augments the inflammatory response including IL-6 [20, 45]. HMGB1 has been proposed to mediate inflammation and nociception in preclinical models or in secondary headaches such as in coronavirus disease 2019 (COVID-19) [21, 22]. HMGB1, NLRP3 and IL-6 levels were positively correlated with headache in patients with COVID-19. Brain HMGB1 level was positively correlated with serum CGRP level and trigeminal pain behavior in our study. Therefore, MOH seems to be accompanied by innate immune response as brain HMGB1 and serum LBP levels were increased.

An association of inflammatory cytokines and migraine was revealed by a recent meta-analysis, that concluded higher levels of pro-inflammatory cytokines IL-6, IL-8 and TNF-α were detected in migraine patients compared to control subjects [18]. Elevated IL-6 levels were shown to be associated with headache chronification in migraine patients [47]. Increased serum IL-6 levels were not only related to headache chronification but also with headache severity and drug unresponsiveness in COVID-19 headache which is also a secondary headache [21, 48]. The significant role of IL-6 as a both pro-inflammatory cytokine and nociceptive molecule in trigeminal system is implicated in preclinical NTG models [17]. IL-17 is a key pro-inflammatory cytokine involved in neuroinflammation, cognitive dysfunction and gastrointestinal disorders such as IBS. IBS symptoms were shown to be higher in MOH patients and subjective cognitive complaints were increased in MOH patients with IBS [49]. Both serum and brain levels of IL-17 were significantly increased in MOH group and were all correlated with biomarkers of leaky gut and pain behavior. Higher levels of cerebral cortical HMGB1 and IL-17 cortex indicate that the inflammatory response in the central nervous system is associated with MOH and may contribute to the maintenance of chronic pain. Besides NTG model [19], NSAID induced MOH is also related to pro-inflammatory cytokine IL-17 increase in the brain. Chronic paracetamol administration has been also shown to increase inflammatory cytokine expressions (IL1-α and TNF-α) in hippocampus however no association with pain was demonstrated in the same study [50].

CGRP is a prime molecule involved in migraine headache signaling and has a role in initiating the local inflammatory response [51]. CGRP levels are found to be high in the systemic circulation in migraine attacks and antagonism of circulating CGRP is a successful treatment in migraine patients [52, 53]. Regarding the above facts, CGRP levels were correlated with trigeminal pain behavior in this MOH model. CGRP also correlated positively with HMGB1 and IL-17 levels in the brain and LBP, VE-cadherin, occludin and IL-17 levels in the serum.

The present study has several limitations. One of the limitations was that an extensive inflammatory response panel including TNF-α and anti-inflammatory cytokines was not studied. Additionally, male rats were not used in the experiments to decide on the sexual dimorphism in piroxicam induced MOH model. The peripheral and central mechanisms of trigeminal nociceptive system involvement remain to be investigated. Another limitation is that the disruption of intestinal barrier in the GI tract was not studied. Translating these findings to humans is necessary and the results remain to be confirmed in patients with MOH.

We showed for the first time that MOH induced by chronic use of oral piroxicam, is linked to leaky gut as LBP, tight junction and adherence junction protein levels in the blood circulation were elevated. We used only female rats to model MOH for clinical relevance as both MOH and chronic migraine patients are predominantly female and NSAIDs have strong perturbations in intestinal permeability in women. All animals were tested outside the estrus and pro-estrus phase, eliminating their confounding effect on trigeminal pain response. Moreover, other strengths of our study are the use of a NSAID for MOH induction since they are the most common analgesics overused in MOH patients and the oral use of piroxicam to increase clinical congruency.

## Conclusion

Chronic oral piroxicam administration for 5 weeks resulted in reduced periorbital mechanical withdrawal thresholds associated with increased head-face grooming, freezing and head shake behavior and elevated serum CGRP levels in female rats all consistent with trigeminal nociception in MOH. MOH is accompanied by elevated serum LBP, occludin and VE-cadherin levels, which suggest the LPS leak from intestinal lumen into the blood circulation and leaky gut. Moreover, a significant inflammatory response in the bloodstream and cerebral cortex was identified in this MOH model. The present study provided evidence linking MOH and leaky gut in female rodents for the first time. Leaky gut must be taken into consideration in patients with MOH for long-term management of this most prevalent secondary headache associated with chronic migraine.

## Abbreviations

CGRP: Calcitonin gene-related peptide
COVID-19: Coronavirus disease 2019
GI: Gastrointestinal
HMGB1: High mobility group box-1
IL: Interleukin
LBP: Lipopolysaccharide binding protein
LPS: Lipopolysaccharides
MOH: Medication overuse headache
NTG: Nitroglycerin
NLRP3: NOD-, LRR- and pyrin domain-containing protein 3
NSAIDS: Nonsteroidal anti-inflammatory drugs
TNF-α: Tumor necrosis factor alpha
VE-cadherin: Vascular endothelial cadherin
